# Conservation of the Threatened Arabian Wolf (*Canis lupus arabs*) in a Mountainous Habitat in Northwestern Saudi Arabia

**DOI:** 10.3390/biology14070839

**Published:** 2025-07-09

**Authors:** Abdulaziz S. Alatawi

**Affiliations:** Department of Biology, Faculty of Science, University of Tabuk, Tabuk 71491, Saudi Arabia; abalatawi@ut.edu.sa

**Keywords:** Al-Lawz mountain, Arabian desert, carnivore, conservation, Tabuk province, threats

## Abstract

The expansion of human activities can degrade natural habitats, thereby increasing threats to wildlife conservation. The wild populations of many species have declined due to the modification of natural habitats by humans. The Arabian wolf (*Canis lupus arabs*) is a subspecies of the gray wolf that is of conservation concern across its distribution range. The Arabian wolf is understudied in certain habitats (e.g., mountainous areas), which limits understanding of its overall ecology. Given its vulnerable conservation status, this study aimed to collect relevant data and information on incidents and potential threats facing this predator in the rugged mountainous habitats of western Tabuk province, Saudi Arabia, and how the effects of these threats can be minimized. In these mountain habitats Arabian wolves encounter various severe threats that challenge relevant conservation efforts. Observations of such threats—some of which result in wolf mortality—represent serious challenges to the survival of wild Arabian wolves. Conflicts with humans and livestock represent considerable threats that must be appropriately managed. Additionally, the potential association between Arabian wolves and free-ranging dogs requires further investigation. Various conservation scenarios and mitigation approaches can be applied to help reduce negative impacts on Arabian wolf populations and maximize their likelihood of survival. Overall, ensuring the persistence of such a unique desert-adapted apex predator in this ecosystem must become a conservation priority.

## Simple Summary

The Arabian wolf (*Canis lupus arabs*) is an understudied carnivore species with a vulnerable conservation status. Thus, gathering information regarding its current status is considered a basic step toward effective conservation activities. Even in arid mountainous habitats, the Arabian wolf encounters various challenges, threats, and conflicts, with some resulting in wolf mortality. Therefore, the long-term survival of this carnivore remains a major concern, especially if existing threats continue and escalate. Focusing more attention on understanding Arabian wolf habitat suitability and managing anthropogenic impacts are crucial steps to prevent a more critical conservation situation for this species.

## 1. Introduction

Natural habitats are not as isolated from anthropogenic disturbances as they used to be since the accessibility and utilization of such habitats by humans have dramatically increased. This implies that the overall habitat suitability for some species might be compromised. Anthropogenic activities are among the top threats affecting wildlife [1,2,3]. Such activities can create changes in the structure of affected habitats, leading to potential reductions in their quantity and quality that can result in threats to the survival of certain species, resulting in an uncertain future [1,2,3,4,5]. The effects of land-use change on species vary depending on their characteristics [6]. Upon encountering threats, certain wild animals might adapt different techniques to ensure their survival and better adjust to new conditions, such as changing their activity time [7] or expanding their foraging search area [8,9]. However, the alternative resources generated by anthropogenic activities can represent an important portion of certain wild animals’ diets [10,11,12]. Moreover, the presence of some wild carnivore species around human centers is becoming normal in many areas [11,13]. However, the presence of wildlife around human activities and their interests might trigger conflicts with humans [14,15]. Notably, human–wildlife conflict can have serious negative consequences for wildlife, which can threaten their conservation status [16,17,18].

The gray wolf (*Canis lupus* Linnaeus, 1758) is a carnivore that experiences human–wildlife conflicts across its distribution range. The gray wolf is a well-known mammalian terrestrial carnivore with a global distribution [19,20,21]. This species occupies various habitats, including forests, grasslands, and even arid deserts [17,18,20,21,22,23]. Since this predator serves an important role in its surrounding habitats, its absence could adversely affect the trophic level of an ecosystem [4,24,25]. Gray wolves’ opportunistic diet and foraging flexibility allow them to consume food that is readily available and easy to acquire [19,26,27]. Despite centuries of population decline due to eradication in most of this species’ distributional range, gray wolf populations seem to be recovering globally [4,19,21,25]. The global International Union for Conservation of Nature (IUCN) Red List assessment indicated that gray wolf populations are experiencing improvement, with a stable global population trend [21]. Hence, the global conservation status for this species is listed as Least Concern [21]. However, even with legal protection throughout most of its range, the gray wolf still faces a variety of serious threats in certain areas of its range, which are largely connected to human activities such as habitat loss and fragmentation, persecution, and human conflict [21,28]. Therefore, the long-term survival of this carnivore in certain localities remains a major concern—especially if these threats continue and escalate [18,22,28].

The Arabian wolf (*Canis lupus arabs* Pocock, 1934) is recognized as a subspecies of the gray wolf [21,29]. This species inhabits the arid environments of the Arabian Peninsula and Southern Levant region, spanning different countries [22,30]. The Arabian wolf is uniquely adapted to live in harsh environments with low productivity, such as arid deserts [22]. It is one of the last remaining apex carnivore predators in its distribution range after other carnivore predators were eradicated from the region [22,31]. Factors such as its diverse diet, foraging behavior, dispersal ability, and large home range contribute to supporting its persistence in the wild—even in habitats where it had previously been extirpated [32]. Despite being protected throughout most of its distribution range, this species faces several key challenges, such as habitat degradation, the low abundance of wild prey, persecution, and human conflict [18,22]. The regional conservation status of the gray wolf in the Arabian Peninsula is listed as Vulnerable [28]. However, one study proposed that the national IUCN status for the Arabian wolf should be listed as Endangered in Saudi Arabia [18]. Overall, this conservation status reflects the difficult situation faced by the Arabian wolf in this challenging environment and geopolitically complicated part of the world [22].

Saudi Arabia constitutes a massive portion of the known spatial distribution of the Arabian wolf [18,22]. Various studies have reported on Arabian wolf distribution in different parts of Saudi Arabia [18,32,33,34,35]. One study updated the Arabian wolf distribution map for Saudi Arabia, showing its wide spatial distribution [32]. The Arabian wolf was also reported to be in many localities across Saudi Arabia based on both early and recent records [18]. Notably, the Arabian wolf is one of the most persecuted carnivore species in Saudi Arabia [18]. For instance, Arabian wolves were reported to be sold in a market in Tabuk province [36], while photographs of their carcasses (e.g., hanging on trees) are documented in several published works from Saudi Arabia [16,18,32,34,35]. Although Aloufi and Amr [34] reported records of wolves in Tabuk province, most records were of dead wolves. Since the Arabian wolf is a poorly studied species, a knowledge gap exists that must be addressed.

**Figure 1:**
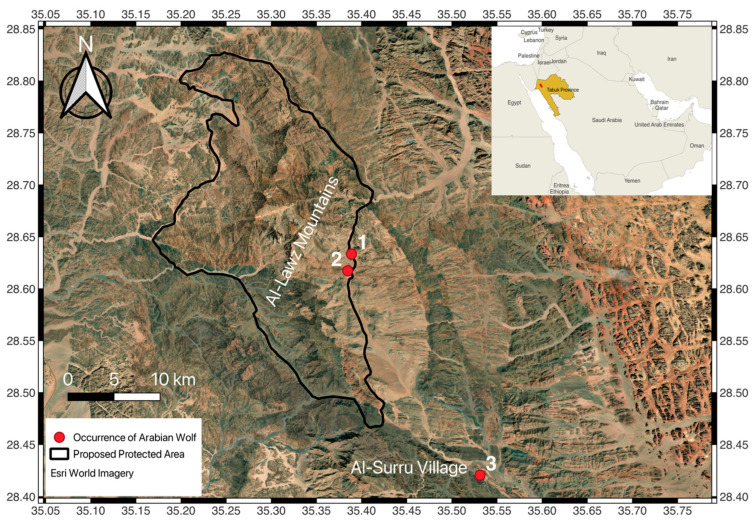
Red points on the map indicate Arabian wolf observations in Al-Lawz mountain habitats (1: vehicle collision; 2: roaming with free-ranging dogs) and in Al-Surru village (3: shot and killed), Tabuk province, Saudi Arabia. The black border line represents the proposed protected area “Jabal al-Lawz” The protected area’s shapefile was downloaded from the Protected Planet website (https://www.protectedplanet.net/en, accessed on 12 March 2025). The study area map was generated using Esri World Imagery in QGIS software (version 3.42.1).

For the Arabian wolf, spatial distribution records and raw data remain far from sufficient or complete, particularly for northern Saudi Arabia. This shortfall raises both short- and long-term conservation concerns. With the growing expansion of human activities and urban sprawl into wild habitats, human presence in remote mountainous habitats has noticeably grown. The human-modified conditions created by such developments could worsen the conservation status of certain wildlife of conservation concern, such as the Arabian wolf. Therefore, this study aims to highlight and discuss various observed threats that can jeopardize the survival of this species in the wild and raise how to possibly minimize their effects. This study specifically focused on a population of Arabian wolves in remote and rugged mountainous habitats located in northwestern Tabuk province, Saudi Arabia, specifically around Al-Lawz mountain and Al-Surru village (Figure 1).

## 2. Materials and Methods

### 2.1. Study Area

The study area is located in the mountainous habitat of northwestern Tabuk province, Saudi Arabia. This area is characterized by rugged mountains with high elevations at certain sites, such as Al-Lawz mountain (2400 m) [37]. The relatively undisturbed natural environment of these mountains, as well as the potential for snowfall during winter on Al-Lawz mountain, have resulted in the area becoming a famous tourist destination. Over the last decade, the presence of humans and their activities have been relatively low. However, with recent infrastructure development and expansion, human presence and construction activity (e.g., for houses, farms, and transportation networks) have increased dramatically in these mountainous habitats. Furthermore, the Al-Lawz mountain area is experiencing ongoing tourism-related megaprojects, resulting in increased human presence and activity in this once-isolated habitat. These modification scenarios can influence the surrounding ecosystem and possibly some native wildlife.

### 2.2. Data Collection

Field visits were conducted from January to May of 2025, in the Al-Lawz mountain and Al-Surru village areas (Figure 1) to collect relevant information and data to understand the conservation status of the Arabian wolf. The threats that Arabian wolves experienced within the visited habitats were recorded. Moreover, potential conservation mechanisms that can help ease the effects of such threats on this species were highlighted. Finally, notes were made on occurrence patterns based on the observed incidents.

## 3. Results

Various threats were recorded during field visits, including some that resulted in wolf mortalities. Conservation threats for the Arabian wolf observed in the visited habitats include potential hybridization with free-ranging dogs, conflict with humans over livestock and resources, and human expansion into their natural habitats (e.g., through establishing road networks). Some recommended conservation strategies and mitigation approaches that can be applied include the activation of a proposed protected area to restore and protect natural habitat, ensuring a sufficient abundance of wild prey species, increasing public awareness, and involving local communities in the conservation process. All observed incidents that represent threats to the Arabian wolf occurred at night and were close to human presence and activities. Notably, no such threats were reported in remote, high-elevation mountainous areas that are far from human presence.

## 4. Discussion

### 4.1. Threats Facing the Arabian Wolf in the Study Area

#### 4.1.1. Potential Hybridization Between Wolves and Free-Ranging Dogs

**Figure 2:**
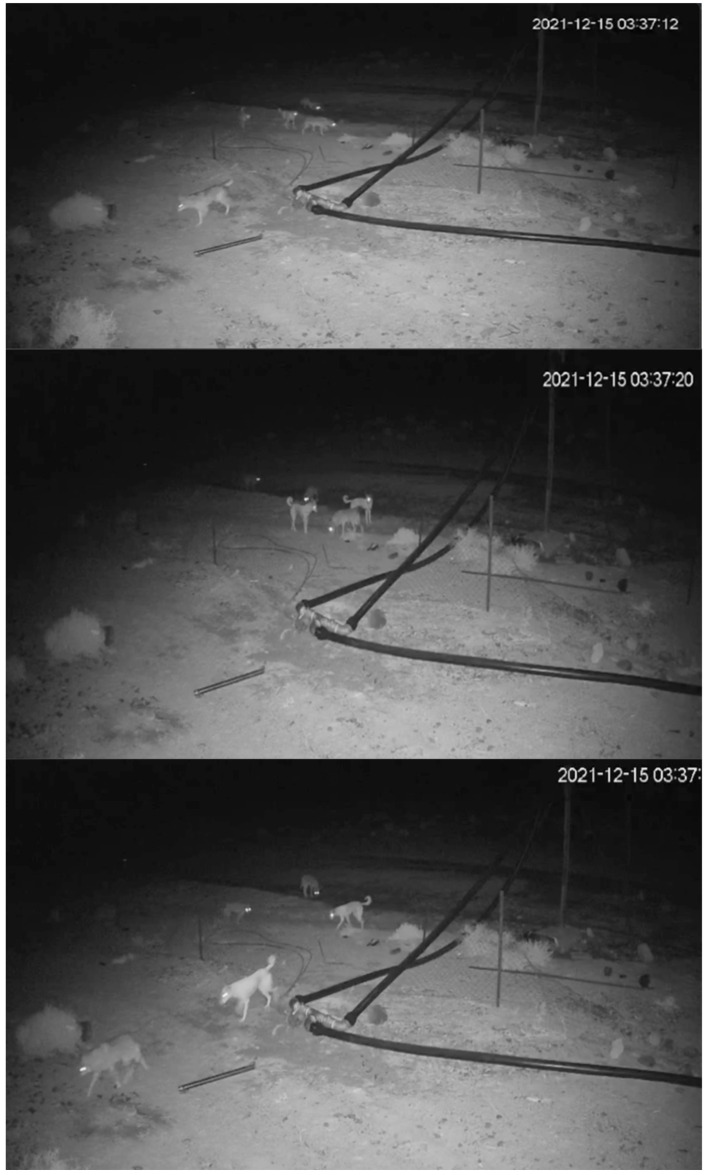
A group of Arabian wolves recorded foraging with free-ranging dogs near a farm fence. Photographs are screenshots taken from a video recorded by a surveillance camera in the Al-Lawz mountain area on 15 December 2021.

**Figure 3:**
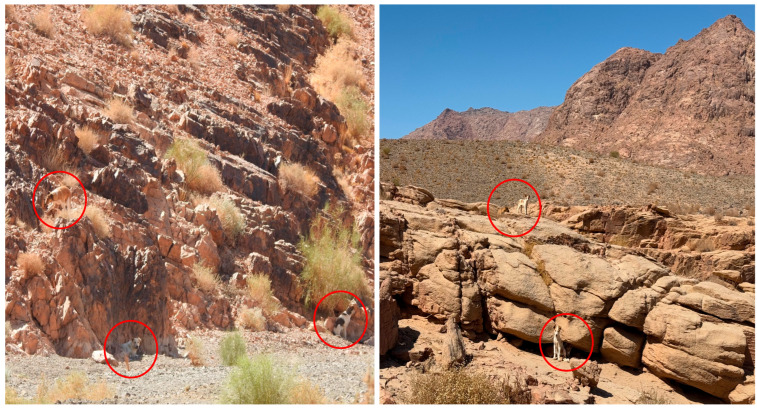
The number of free-ranging dogs is noticeably increasing in this wild mountainous habitat. Photographs were taken in Al-Surru village and the Al-Lawz mountain area in 2025. Photographs by Abdulaziz Alatawi.

Hybridization can occur between wolves and dogs (*Canis lupus familiaris*), which can produce hybrid individuals [38,39,40]. Small wolf populations that are in close contact with free-ranging dogs are more vulnerable to hybridizing with them [39]. Wolf–dog hybridization can exacerbate conservation concerns surrounding wolves and their genetic integrity [38,39,40,41,42,43]. Notably, such hybridization is assumed to change the behavior of hybrid wolves [44]. In 2021, a surveillance camera placed on a farm fence in the Al-Lawz mountain area recorded a group of wolves roaming with a group of dogs at night (Figure 2). From a conservation perspective, such documentation is extremely important to consider thoroughly. Further scientific investigations in the field should be conducted, even if no signs of hybridization were visible in this group. Notably, the potential association between Arabian wolves and free-ranging dogs has previously been observed in central Saudi Arabia [45]. In the Al-Lawz mountain area and Al-Surru village, the noticeable presence of free-ranging dogs was not common years ago. However, their presence has recently increased dramatically due to their increased use as guard dogs by humans. As a result, the number of free-ranging dogs has also increased in these habitats (Figure 3). However, the extent of hybridization in this region remains unknown [46], and the effects of hybridization on Arabian wolves might take some time to appear [45]. Importantly, the presence of free-ranging dogs must be properly investigated to determine whether they pose a conservation threat to wolves through hybridization, competition, and disease transmission [39,43].

#### 4.1.2. Human–Wolf Conflict

**Figure 4:**
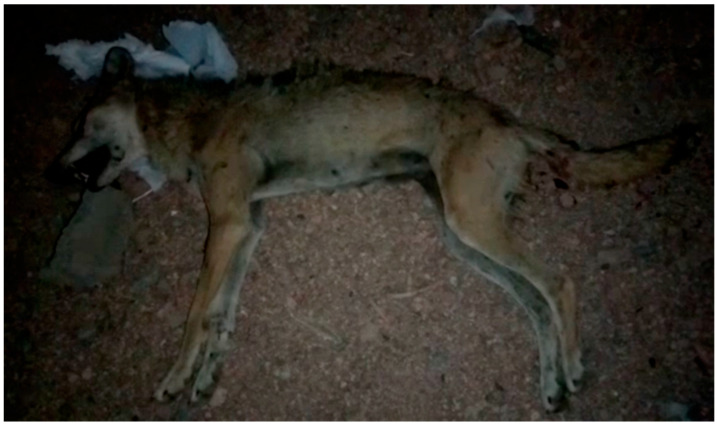
An Arabian wolf was shot and killed in the backyard of a house after attacking its residents. The incident occurred in Al-Surru village in 2021.

Human–wildlife conflict is a significant challenge that cannot be overlooked when aiming to conserve wild animals [47,48,49]. Paying more attention to this type of conflict is especially important when dealing with endangered or vulnerable species. Given the scarcity and depletion of their wild prey, wolves might roam further to hunt for food resources (e.g., livestock and human food waste) that can exist near human activities, eventually leading to potential conflicts [17,26,50]. Poaching and retaliatory killings are known consequences of human–wolf conflicts [17,50,51]. In 2021, local news reported that a wolf was shot and killed in the backyard of a local house in Al-Surru village after attacking the home’s residents (Figure 4). Another important event that might trigger a conflict resulting in the shooting of wolves is their depredation of livestock. Domestic species and livestock can represent an important food source for wolves, especially in light of the paucity of wild prey species [17,19,26,27]. The shooting and killing of wolves still occur near human settlements in these areas. Notably, such shootings are more likely to occur during attempts to protect livestock from potential wolf attacks. In the mountainous habitats of Tabuk province, reports of human–wolf conflicts over livestock resulting in wolf mortality are not a new issue [16]. Wolf–livestock conflict is considered one of the most substantial threats to Arabian wolves in this region, where pastoralism and livestock farming remain common practices [16,22]. These shooting mortality scenarios highlight why the presence of wild carnivores near human interests is more likely perceived by some community members as a direct threat that must be immediately eliminated.

#### 4.1.3. Human Expansion into Natural Habitats

**Figure 5:**
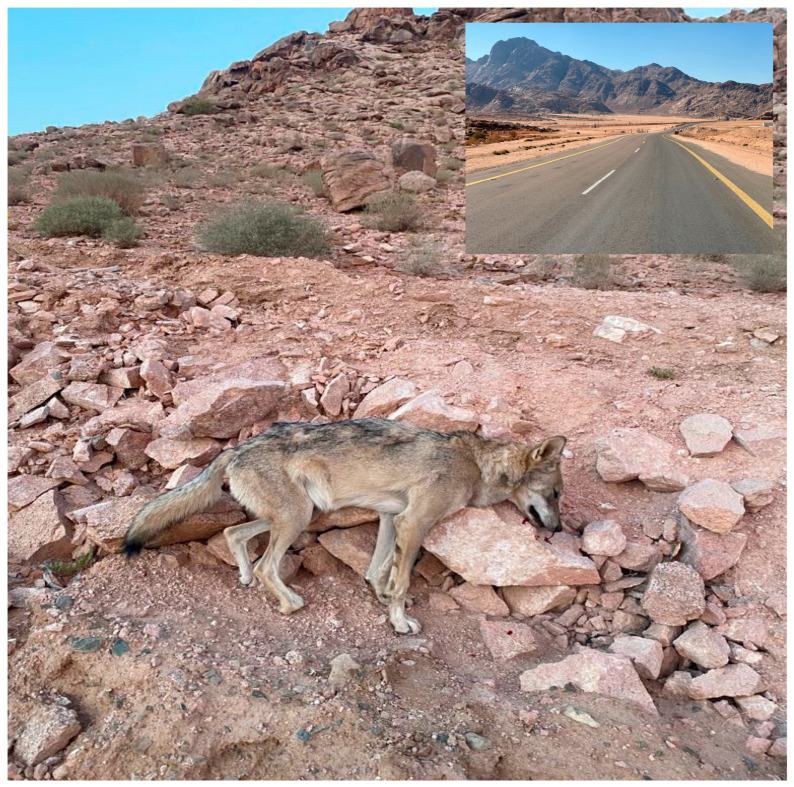
An Arabian wolf was found dead next to a newly paved road (see inset map). The death of this wolf most likely occurred due to a vehicle collision. The incident occurred in the Al-Lawz mountain area in 2023. Photograph by Omar Alatawi.

The expansion of human activities implies that more natural habitats will be lost and/or modified in favor of human interests. In the Arabian Peninsula, wild carnivore species have been significantly affected by habitat loss due to human activities [28,46]. Notably, the development of road networks can fragment natural habitats and increase the mortality rates of many wild species through vehicle collisions [8,52]. In response, some species have learned to adapt to navigate roads in human-modified habitats [52,53]. The areas surrounding the Al-Lawz mountains and Al-Surru village have recently been experiencing massive infrastructure developments and tourism activities. In 2023, an Arabian wolf was found dead at dawn next to a newly established paved road in the Al-Lawz mountain area (Figure 5). The cause of death for this individual can most likely be attributed to a vehicle collision. This paved road is part of a road network that is being established to serve and supply nearby tourism projects. The establishment of roads in remote mountainous habitats, as well as the subsequent presence of humans and their associated traffic flow, will likely lead to the increased disturbance of local wildlife in their natural habitats.

### 4.2. Arabian Wolf Conservation Approaches

The roles that Arabian wolves serve in their native ecosystems are important [31]. Notably, the small size of the Arabian wolf population makes this unique carnivore even more vulnerable to disturbances [22]. Even in understudied remote mountainous habitats, various challenges, threats, and conflicts can still inevitably occur. Consequently, certain conservation efforts and mitigation strategies can be applied to address both direct and indirect threats and effectively minimize their influence.

The restoration and conservation of natural habitats that Arabian wolves depend on is a mandatory process [16,18,22]. The minimization and restriction of human disturbance by regulating human activities in natural habitats must be prioritized and enforced to achieve positive conservation outcomes. Establishing protected areas can also aim to fulfill this conservation demand since these areas have demonstrated their effectiveness in conserving vulnerable species [16,54]. Notably, the Arabian wolf has been recorded in different protected areas throughout Saudi Arabia [32,33,55]. Thus, protected areas appear to serve a vital and direct role in Arabian wolf conservation efforts [22,32]. According to the Protected Planet website, a proposed protected area named “Jabal al-Lawz” is planned to be established around the habitats of Al-Lawz mountain (Figure 1), with a reported total size of 489.82 km^2^ [56]. In parallel with protected area efforts, conservation outside of protected areas is also necessary for such carnivores to achieve good conservation outcomes due to their large home ranges and dispersal abilities.

Non-lethal mitigation strategies can also be followed to reduce wolves’ predation on livestock [43]. First, ensuring a sufficient abundance of wild prey (i.e., food availability) and avoiding their depletion can reduce conflict with humans over wolves preying on livestock and other domestic species [17,19,50]. It has been shown that wolves might prey less upon domestic animals when wild prey is available [19,26]. Second, despite the threats that dogs may pose to wolves, their use as guard dogs can be highly beneficial by lowering the rate of wolf predation on livestock [39,57]. Third, improving livestock husbandry practices can considerably reduce cases of wolf predation on livestock [19,50]. During the field visits, it was observed that the construction of some livestock housings/pens was inadequate to deter wolf attacks. Furthermore, wolf–livestock conflict can also be lessened by implementing a compensation program for livestock losses [17,43]. Overall, utilizing non-lethal techniques can support more peaceful coexistence and avoid the poaching and retaliatory killing of wolves.

Public awareness and educational programs also serve a crucial role in achieving positive conservation outcomes [25,58]. Understanding the factors affecting people’s attitudes and perceptions surrounding wolves is important [59,60]. Systematic campaigns (e.g., in schools and social gatherings) to increase social acceptance of the Arabian wolf and promote knowledge of its importance can greatly assist in its conservation efforts. For instance, establishing a special event dedicated to Arabian wolves—similar to the special event dedicated to increasing awareness about the Arabian leopard (*Panthera pardus nimr*) (e.g., International Day of the Arabian Leopard)—would likely garner more public attention to its importance, conservation efforts, and status. Additionally, employing local community members who live near Arabian wolf habitats (e.g., as rangers) can also assist in conservation efforts by reporting wolf observations, incidents, threats, and any other relevant information. Overall, such reports can help us understand more about their ecology, distribution patterns, and possible threats. Lastly, social awareness campaigns must also address the lack of knowledge regarding potential wolf–dog hybridization and how this could negatively impact conservation efforts [40].

### 4.3. Arabian Wolf Distribution in the Study Area

Arabian wolves have large home ranges and can cover wide areas when hunting for food. The distribution and activity patterns of wolves are affected by various interacting factors [61,62]. Notably, human-dominated landscapes and ecosystem modification could change the consumption behavior of wolves [63]. In the present study, all recorded threats and incidents occurred at night and close to human presence and activities, indicating the potential reliance of Arabian wolves on anthropogenic food resources. The presence of rich, easy-to-acquire food such as waste, livestock, and animal carcasses encourages wolves to visit these areas and search for food. This foraging pattern is especially emphasized in light of the low abundance and density of wild prey species [26]. In the Al-Lawz mountain area, four wolves were observed feeding on a dead camel near a farm (personal communication with a local resident). Notably, anthropogenic food resources could sustain wolf populations [27]. Wolf diets have been found to include large quantities of livestock [27,64] and even other carnivorous species [63]. Foraging near human activities and potential dependence on anthropogenic food resources might be considered an alarming sign for the conservation of this apex predator in the study area.

## 5. Conclusions

To avoid reaching a critical conservation situation, the Arabian wolf may require site- and species-specific protection efforts to address its conservation needs [22]. Since Saudi Arabia represents a massive proportion of the Arabian wolf’s overall spatial distribution [22,32], the conservation of this threatened species is under consideration [18]. According to Al Ahmari et al. [18], more than 40 wolves are in sheltering centers awaiting rewilding in Saudi Arabia. The Arabian wolf is an officially protected species in Saudi Arabia [65]. Thus, hunting an individual Arabian wolf can result in a fine of up to SAR 80,000 (USD 21,333) [65]. Such executive regulations for hunting wildlife are in place to deter the illegal hunting of vulnerable species. Importantly, systematic field visits will be required to monitor the population size, habitat suitability, and food availability of the Arabian wolf to assist in establishing reliable raw data that can be used to fill existing knowledge gaps and aid in decision-making processes. To this end, given the existing conservation challenges faced by this species, moving conservation practices forward is strongly advised [16,18,43,54], with cross-border collaboration being necessary to protect this important predator [22].

## Data Availability

Data available upon request.

## References

[1] Newbold T., Hudson L.N., Hill S.L., Contu S., Lysenko I., Senior R.A., Börger L., Bennett D.J., Choimes A., Collen B. (2015). Global effects of land use on local terrestrial biodiversity. Nature.

[2] Ripple W.J., Newsome T.M., Wolf C., Dirzo R., Everatt K.T., Galetti M., Hayward M.W., Kerley G.I., Levi T., Lindsey P.A. (2015). Collapse of the world’s largest herbivores. Sci. Adv..

[3] Tilman D., Clark M., Williams D.R., Kimmel K., Polasky S., Packer C. (2017). Future threats to biodiversity and pathways to their prevention. Nature.

[4] Ripple W.J., Estes J.A., Beschta R.L., Wilmers C.C., Ritchie E.G., Hebblewhite M., Berger J., Elmhagen B., Letnic M., Nelson M.P. (2014). Status and ecological effects of the world’s largest carnivores. Science.

[5] Ceballos G., Ehrlich P.R., Raven P.H. (2020). Vertebrates on the brink as indicators of biological annihilation and the sixth mass extinction. Proc. Natl. Acad. Sci. USA.

[6] Newbold T., Bentley L.F., Hill S.L., Edgar M.J., Horton M., Su G., Şekercioğlu Ç.H., Collen B., Purvis A. (2020). Global effects of land use on biodiversity differ among functional groups. Funct. Ecol..

[7] Gil-Fernández M., Harcourt R., Newsome T., Towerton A., Carthey A. (2020). Adaptations of the red fox (*Vulpes vulpes*) to urban environments in Sydney, Australia. J. Urban Ecol..

[8] Alatawi A.S. (2024). Role of Agricultural Areas as Shelters for Carnivores in a Desert Ecosystem in Saudi Arabia. Pak. J. Zool..

[9] Wolf C., Ripple W.J. (2016). Prey depletion as a threat to the world’s large carnivores. R. Soc. Open Sci..

[10] Contesse P., Hegglin D., Gloor S., Bontadina F., Deplazes P. (2004). The diet of urban foxes (*Vulpes vulpes*) and the availability of anthropogenic food in the city of Zurich, Switzerland. Mamm. Biol..

[11] Bateman P.W., Fleming P.A. (2012). Big city life: Carnivores in urban environments. J. Zool..

[12] Handler A.M., Lonsdorf E.V., Ardia D.R. (2020). Evidence for red fox (*Vulpes vulpes*) exploitation of anthropogenic food sources along an urbanization gradient using stable isotope analysis. Can. J. Zool..

[13] Plumer L., Davison J., Saarma U. (2014). Rapid Urbanization of Red Foxes in Estonia: Distribution, Behaviour, Attacks on Domestic Animals, and Health-Risks Related to Zoonotic Diseases. PLoS ONE.

[14] Soulsbury C.D., White P.C. (2015). Human–wildlife interactions in urban areas: A review of conflicts, benefits and opportunities. Wildl. Res..

[15] Schell C.J., Stanton L.A., Young J.K., Angeloni L.M., Lambert J.E., Breck S.W., Murray M.H. (2021). The evolutionary consequences of human–wildlife conflict in cities. Evol. Appl..

[16] Alatawi A.S. (2022). Conservation action in Saudi Arabia: Challenges and opportunities. Saudi J. Biol. Sci..

[17] Hamid A., Mahmood T., Fatima H., Hennelly L.M., Akrim F., Hussain A., Waseem M. (2019). Origin, ecology and human conflict of gray wolf (*Canis lupus*) in Suleman Range, South Waziristan, Pakistan. Mammalia.

[18] Al Ahmari A., Neyaz F., Shuraim F., Al Ghamdi A., Al Boug A., Alhlafi M., Al Jbour S., Angelici F.M., Alaamri S., Al Masabi K. (2025). Diversity and Conservation of Carnivores in Saudi Arabia. Diversity.

[19] Newsome T.M., Boitani L., Chapron G., Ciucci P., Dickman C.R., Dellinger J.A., López-Bao J.V., Peterson R.O., Shores C.R., Wirsing A.J. (2016). Food habits of the world’s grey wolves. Mammal Rev..

[20] Wang L., Ma Y.-P., Zhou Q.-J., Zhang Y.-P., Savolainen P., Wang G.-D. (2016). The geographical distribution of grey wolves (*Canis lupus*) in China: A systematic review. Zool. Res..

[21] Boitani L., Phillips M., Jhala Y. (2023). *Canis lupus* (Amended Version of 2018 Assessment). The IUCN Red List of Threatened Species 2023: E.T3746A247624660. https://www.iucnredlist.org/species/3746/247624660.

[22] Bonsen G.T., Wallach A.D., Ben-Ami D., Keynan O., Khalilieh A., Dahdal Y., Ramp D. (2024). Navigating complex geopolitical landscapes: Challenges in conserving the endangered Arabian wolf. Biol. Conserv..

[23] Cohen O., Barocas A., Geffen E. (2013). Conflicting management policies for the Arabian wolf *Canis lupus arabs* in the Negev Desert: Is this justified?. Oryx.

[24] Ripple W.J., Beschta R.L. (2012). Trophic cascades in Yellowstone: The first 15 years after wolf reintroduction. Biol. Conserv..

[25] Villeneuve K.A., Proulx G., Proulx G. (2024). Ecological advantages of grey wolf (*Canis lupus*) reintroductions and recolonizations in North America. Wildlife Conservation and Management in the 21st Century-Issues, Solutions, and New Concepts.

[26] Zlatanova D., Ahmed A., Valasseva A., Genov P. (2014). Adaptive diet strategy of the wolf (*Canis lupus* L.) in Europe: A review. Acta Zool. Bulg..

[27] Mohammadi A., Kaboli M., Sazatornil V., Lopez-Bao J.V. (2019). Anthropogenic food resources sustain wolves in conflict scenarios of Western Iran. PLoS ONE.

[28] Mallon D.P., Hilton-Taylor C., Amori G., Baldwin R., Bradshaw P.L., Budd K. (2023). The Conservation Status and Distribution of the Mammals of the Arabian Peninsula.

[29] Bray T.C., Mohammed O.B., Butynski T.M., Wronski T., Sandouka M.A., Alagaili A.N. (2014). Genetic variation and subspecific status of the grey wolf (*Canis lupus*) in Saudi Arabia. Mamm. Biol..

[30] Eid E., Abu Baker M., Amr Z. (2020). National Red Data Book of Mammals in Jordan.

[31] Bonsen G.T., Wallach A.D., Ben-Ami D., Keynan O., Khalilieh A., Shanas U., Wooster E.I., Ramp D. (2022). Tolerance of wolves shapes desert canid communities in the Middle East. Glob. Ecol. Conserv..

[32] Cunningham P.L., Wronski T. (2010). Arabian Wolf Distribution Update from Saudi Arabia. Canid News.

[33] Wronski T., Macasero W. (2008). Evidence for the persistence of Arabian Wolf (*Canis lupus pallipes*) in the Ibex Reserve, Saudi Arabia and its preferred prey species. Zool. Middle East.

[34] Aloufi A.A., Amr Z.S. (2018). Carnivores of the Tabuk Province, Saudi Arabia (Carnivora: Canidae, Felidae, Hyaenidae, Mustelidae). Lynx New Ser..

[35] Zafar-ul Islam M., Boug A., Shehri A., da Silva L.G. (2019). Geographic distribution patterns of melanistic Arabian Wolves, *Canis lupus arabs* (Pocock), in Saudi Arabia (*Mammalia*: *Carnivora*). Zool. Middle East.

[36] Aloufi A., Eid E. (2014). Conservation perspectives of illegal animal trade at markets in Tabuk, Saudi Arabia. TRAFFIC Bull..

[37] Al Saud M.M. (2020). Sustainable Land Management for NEOM Region.

[38] Randi E. (2011). Genetics and conservation of wolves *Canis lupus* in Europe. Mammal Rev..

[39] Lescureux N., Linnell J.D. (2014). Warring brothers: The complex interactions between wolves (*Canis lupus*) and dogs (*Canis familiaris*) in a conservation context. Biol. Conserv..

[40] Donfrancesco V., Ciucci P., Salvatori V., Benson D., Andersen L.W., Bassi E., Blanco J.C., Boitani L., Caniglia R., Canu A. (2019). Unravelling the Scientific Debate on How to Address Wolf-Dog Hybridization in Europe. Front. Ecol. Evol..

[41] Randi E. (2008). Detecting hybridization between wild species and their domesticated relatives. Mol. Ecol..

[42] Werhahn G., Senn H., Macdonald D.W., Sillero-Zubiri C. (2022). The Diversity in the Genus *Canis* Challenges Conservation Biology: A Review of Available Data on Asian Wolves. Front. Ecol. Evol..

[43] Werhahn G., Augugliaro C., Kabir M., Hennelly L.M., Chetri M., Al Hikmani H., Mohammadi A., Jhala Y.V., Macdonald D.W., Farhadinia M.S. (2025). Asia’s Wolves and Synergies with Big Cats. Conserv. Lett..

[44] Herzog S. (2018). Return of grey wolf (*Canis lupus*) to Central Europe: Challenges and recommendations for future management in cultural landscapes. Ann. For. Res..

[45] Barichievy C., Clugston S., Sheldon R. (2017). Association between an Arabian wolf and a domestic dog in central Saudi Arabia. Canid Biol. Conserv..

[46] Mallon D., Budd K. (2011). Regional Red List Status of Carnivores in the Arabian Peninsula.

[47] König H.J., Ceaușu S., Reed M., Kendall H., Hemminger K., Reinke H., Ostermann Miyashita E.F., Wenz E., Eufemia L., Hermanns T. (2021). Integrated framework for stakeholder participation: Methods and tools for identifying and addressing human–wildlife conflicts. Conserv. Sci. Pract..

[48] Basak S.M., Rostovskaya E., Birks J., Wierzbowska I.A. (2023). Perceptions and attitudes to understand human-wildlife conflict in an urban landscape—A systematic review. Ecol. Indic..

[49] Lazure L., Weladji R.B. (2024). Methods to mitigate human–wildlife conflicts involving common mesopredators: A meta-analysis. J. Wildl. Manag..

[50] Janeiro-Otero A., Newsome T.M., Van Eeden L.M., Ripple W.J., Dormann C.F. (2020). Grey wolf (*Canis lupus*) predation on livestock in relation to prey availability. Biol. Conserv..

[51] Montanheiro Paolino R., Testa Jose C., Fernandes-Santos R.C., Bueno Landis M., Medeiros de Pinho G., Medici E.P. (2024). Poaching and hunting, conflicts and health: Human dimensions of wildlife conservation in the Brazilian Cerrado. Front. Conserv. Sci..

[52] Baker P.J., Dowding C.V., Molony S.E., White P.C., Harris S. (2007). Activity patterns of urban red foxes (*Vulpes vulpes*) reduce the risk of traffic-induced mortality. Behav. Ecol..

[53] Kobryn H.T., Swinhoe E.J., Bateman P.W., Adams P.J., Shephard J.M., Fleming P.A. (2023). Foxes at your front door? Habitat selection and home range estimation of suburban red foxes (*Vulpes vulpes*). Urban Ecosyst..

[54] Barichievy C., Sheldon R., Wacher T., Llewellyn O., Al-Mutairy M., Alagaili A. (2018). Conservation in Saudi Arabia; moving from strategy to practice. Saudi J. Biol. Sci..

[55] Seddon P.J., van Heezik H., Nader I.A. (1997). Mammals of the Harrat al-Harrah Protected Area, Saudi Arabia. Zool. Middle East.

[56] UNEP-WCMC Protected Area Profile for Jabal al-Lawz from the World Database on Protected Areas, March 2025. www.protectedplanet.net.

[57] Marker L.L., Dickman A.J., Macdonald D.W. (2005). Perceived effectiveness of livestock-guarding dogs placed on Namibian farms. Rangel. Ecol. Manag..

[58] Wu Y., Xie L., Huang S.L., Li P., Yuan Z., Liu W. (2018). Using social media to strengthen public awareness of wildlife conservation. Ocean Coast. Manag..

[59] Sakurai R., Tsunoda H., Enari H., Siemer W.F., Uehara T., Stedman R.C. (2020). Factors affecting attitudes toward reintroduction of wolves in Japan. Glob. Ecol. Conserv..

[60] Barmoen M., Bærum K.M., Mathiesen K.E. (2024). Living with wolves: A worldwide systematic review of attitudes. Ambio.

[61] Theuerkauf J. (2009). What Drives Wolves: Fear or Hunger? Humans, Diet, Climate and Wolf Activity Patterns. Ethology.

[62] Llaneza L., López-Bao J.V., Sazatornil V. (2012). Insights into wolf presence in human-dominated landscapes: The relative role of food availability, humans and landscape attributes. Divers. Distrib..

[63] Martins I., Krofel M., Mota P.G., Álvares F. (2020). Consumption of carnivores by wolves: A worldwide analysis of patterns and drivers. Diversity.

[64] Torres R.T., Silva N., Brotas G., Fonseca C. (2015). To Eat or Not to Eat? The Diet of the Endangered Iberian Wolf (*Canis lupus signatus*) in a Human-Dominated Landscape in Central Portugal. PLoS ONE.

[65] Ministry of Environment, Water and Agriculture Executive Regulations for Hunting Wildlife. https://www.mewa.gov.sa/ar/InformationCenter/DocsCenter/RulesLibrary/Pages/default.aspx.

